# Rising CA-125 (Cancer Antigen 125) Levels: Cancer Recurrence or a Vaccine Reaction?

**DOI:** 10.7759/cureus.34534

**Published:** 2023-02-02

**Authors:** Jasmita Parkash, Varinder Bansro, Gurdeep S Chhabra, Zainab Mujahid

**Affiliations:** 1 Internal Medicine, Dr. Rajendra Prasad Government Medical College, Kangra, IND; 2 Internal Medicine, University of Maryland Capital Region Health, Largo, USA; 3 Hematology and Medical Oncology, University Of Maryland Capital Region Health, Largo, USA

**Keywords:** mrna-covid-19 vaccine, tumor marker ca-125, elevated ca-125, ovarian cancer recurrence, covid 19

## Abstract

Cancer antigen 125 (CA-125) is a transmembrane glycoprotein, and it is known to be an essential biomarker in detecting treatment response and recurrence of ovarian cancer. It may also be used in monitoring colorectal cancer. It tends to rise in states of inflammation. Recent studies have demonstrated a temporary rise in CA-125 levels and other cancer biomarkers in patients suffering from coronavirus disease 2019 (COVID-19) infection. However, in the following case report, we hope to shed light on a possible association between CA-125 levels and the COVID-19 mRNA vaccine. We present the case of a 79-year-old woman with moderately differentiated adenocarcinoma of the right adnexa, who had a transient increase in CA-125 levels after a period, during which she underwent treatment for COVID-19 infection and received the first dose of COVID-19 mRNA (Pfizer-BioNTech) vaccine with no evidence of disease progression on imaging.

## Introduction

Cancer antigen 125 (CA-125) is a transmembrane glycoprotein that is expressed on surfaces derived from coelomic epithelium including the female reproductive tract, respiratory tract, and ocular surfaces [1]. CA-125 is an essential biomarker for detecting response to treatment and recurrence of ovarian malignancy [2]. It may also be used in monitoring treatment response in colorectal cancers [2]. However, due to its limited specificity, its usage as a diagnostic marker is restricted. CA-125 levels are elevated in a variety of non-ovarian malignancies such as cervical, colorectal, endometrial, and lung cancers [3]. They can also be elevated in benign conditions involving the coelomic epithelium, such as ovarian cysts, endometriosis, pelvic inflammatory disease, and uterine fibroids along with lung conditions such as pulmonary tuberculosis and chronic obstructive pulmonary disease (COPD) [2]. CA-125 is a marker molecule that is secreted in response to inflammatory cytokines such as interleukin 1 beta, tumor necrosis factor-alpha (TNF-α), and lipopolysaccharide (LPS) [2,3]. The exact mechanism that causes this increase is not well-understood. Its tendency to increase in states of inflammation explains why elevated CA-125 levels can be seen in conditions such as COPD, pelvic inflammatory disease, etc. [2,3].

Recent studies have shown that an increase in CA-125 levels and other cancer biomarkers is seen in patients suffering from severe coronavirus disease 2019 (COVID-19) infection [4]. A retrospective study by Smith et al. has also highlighted the importance of ruling out COVID-19 as a potential etiology for elevated CA-125 levels in patients with ovarian and other gynecological malignancies [2]. The study suggested that severe COVID-19 infection was associated with a transient rise and fall in CA-125 biomarker levels in women with advanced ovarian malignancy [2]. In that scenario, the transient spike of CA-125 was not representative of cancer recurrence as no radiographic evidence of disease progression was present and COVID-19 was determined to be the most likely etiology [2].

The impact of COVID-19 mRNA vaccines on CA-125 levels in patients with gynecological malignancies is a topic that requires further investigation and research. In this report, we present the case of a woman with moderately-differentiated adenocarcinoma of the right adnexa, who had a transient increase in CA-125 levels after a period during which she underwent treatment for COVID-19 infection and received the first dose of COVID-19 mRNA (Pfizer-BioNTech) vaccine with no evidence of disease progression on imaging. Further investigation is required as to whether the COVID-19 infection itself or the mRNA vaccine led to the transient rise in CA-125 levels in her case.

## Case presentation

The patient was a 79-year-old woman with a history of moderately-differentiated adenocarcinoma of the right adnexal region (excised successfully in April 2017). The pathology cells of the excised right adnexal mass showed immunostaining for cytokeratin 7 (CK7), CA-125, mammaglobin, and epithelial cadherin (E Cadherin P1), and frontal nuclear staining was positive for paired box gene 8 (PAX-8), estrogen, and progesterone. The immunostaining was highly suggestive of a primary gynecological malignancy (endometrial vs. ovarian). Since the excision, the patient had been periodically following up with her oncologist and generally doing well. Unfortunately, she was infected with COVID-19 and was admitted to the hospital in January 2022. She received her first dose of the Pfizer-BioNTech COVID-19 mRNA vaccine on February 10, 2022, and the second dose was received on March 24, 2022.

**Figure 1:**
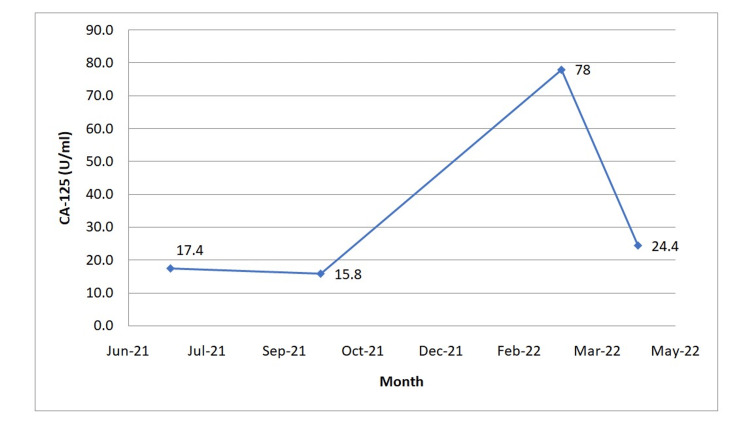
CA-125 levels over a period of one year (June 2021-April 2022)

At her follow-up visit on March 7, 2022, routine blood investigations were performed to check her CA-125 levels. Her CA-125 levels had risen to a level of 78.0 U/ml. The level had been within normal limits at her previous visit on October 4, 2021 (Figure 1, Table 1). The previous CT scan (without contrast) of the abdomen-pelvis region performed in October 2020 had shown no pelvic recurrence of metastatic disease. Her rising CA-125 levels raised the suspicion of a possible malignancy recurrence and, hence, repeat CA-125 levels and positron emission tomography scan with CT (PET/CT) were ordered for restaging purposes. At her one-month follow-up visit in April 2022, her results showed that the CA-125 levels had returned to the normal range of 24.4 U/ml. Her PET/CT performed on April 21, 2022, failed to reveal any uptake in the pelvic region, showing no signs of a possible recurrence of malignancy. At this stage, routine monitoring of CA-125 levels and follow-up were planned.

**Table 1:** CA-125 levels

CA-125 (U/mL)				
Normal Reference Range	June 2021	Oct-21	March 2022	April 2022
0-35	17.4	15.8	78	24.4

The cause of this temporary rise in CA-125 levels is unclear. It could either be the patient’s recent hospitalization for COVID-19 infection or her recent COVID-19 mRNA vaccination.

## Discussion

The COVID-19 pandemic has had a detrimental impact on patients all over the world and the development and distribution of mRNA COVID-19 vaccines have primarily aimed to benefit the public. We presented the case of a woman with a history of excised right adnexal moderately differentiated adenocarcinoma with recent COVID-19 vaccination and hospitalization for COVID-19 infection, showing a transient rise and fall of CA-125 tumor marker levels with no radiographic evidence of disease progression or recurrence; this report highlights the importance of considering both recent COVID-19 vaccination and COVID-19 infection as possible etiologies of rising CA-125 levels once the possibility of malignancy recurrence has been successfully ruled out.

This report supports the findings of a recent study by Johnson et al., which described a transient rise and fall in CA-125 levels in a patient treated for COVID-19 infection with a history of unresectable high-grade serous ovarian carcinoma [2]. This case indicates that the rise in CA-125 levels may not necessarily be an inflammatory response of the body to the COVID-19 infection; it may be an inflammatory response to the COVID-19 mRNA vaccine instead.

It is possible that this temporary rise in CA-125 levels is a vaccine-associated reaction, similar to some other well-known side effects such as pain, swelling, and local lymphadenopathy [4,5]. Lam et al. have presented a similar scenario in a recently published JAMA study [5]. A patient with biopsy-proven ductal carcinoma in situ (DCIS) was found to have axillary lymphadenopathy on MRI [5]. She had received her second dose of the COVID-19 mRNA vaccine a day before undergoing MRI and, after one week, the lymphadenopathy on MRI resolved on its own [5]. The sentinel biopsy was also negative, confirming the diagnosis of COVID-19 vaccine-associated reactive lymphadenopathy [5]. In the field of oncology, such after-effects of COVID-19 vaccines may sometimes be misinterpreted as cancer recurrence and lead to further investigations and procedures [6,7]. However, based on this case report, this brief rise in CA-125 levels may also be a vaccine-associated inflammatory response.

## Conclusions

Our case report highlights the importance of ruling out recent COVID-19 infection and COVID-19 mRNA vaccination as possible etiologies for patients with a rise in CA-125 levels from their baseline along with diligently monitoring for any evidence of disease recurrence or progression through extensive radiological imaging. Further investigations into the possible effects of COVID-19 mRNA vaccinations and COVID-19 itself on CA-125 levels in patients with ovarian and other gynecological malignancies are warranted.
